# A Comprehensive DNA Barcode Reference Library for the Macroinvertebrates of Scottish Seagrass Beds Using Oxford Nanopore Flongle Flowcells

**DOI:** 10.1002/ece3.72219

**Published:** 2025-09-28

**Authors:** E. G. Ross, S. B. Piertney, J. D. Sigwart, N. F. Crook, A. Moreau, K. K. S. Layton

**Affiliations:** ^1^ School of Biological Sciences University of Aberdeen Aberdeen UK; ^2^ Marine Zoology Department, Senckenberg Research Institute and Museum Frankfurt Germany; ^3^ Department of Biology University of Toronto Mississauga Mississauga Canada

**Keywords:** biodiversity studies, DNA barcoding, marine invertebrates, Oxford nanopore sequencing, seagrass habitats

## Abstract

Oxford Nanopore Sequencing Technology (ONT) has emerged as a scalable method for generating DNA barcode reference libraries, capable of sequencing hundreds of DNA barcodes simultaneously using the portable, benchtop MinION sequencing device. In this study, we use ONT Flongle flowcells to produce DNA barcodes for 146 seagrass‐associated marine invertebrate OTUs collected from four seagrass beds in Scotland, targeting COI and 18S V4 regions. We make use of degenerate and group‐specific primer pairs to improve recovery and demonstrate how mapping ONT reads to pre‐existing DNA barcodes can be used to reduce ambiguous basecalls and improve recovery of sequences from contaminated specimens. Overall, this study informs prospective users intending to carry out multimarker DNA barcoding projects using Oxford Nanopore Sequencing. Furthermore, we generated the first DNA barcode reference library for seagrass beds in Scotland to support future biomonitoring of these priority habitats.

## Graphical abstract

In this study we use ONT Flongle Flowcells to produced DNA barcodes for 146 seagrass associated marine invertebrate OTUs collected from four seagrass beds in Scotland, targeting COI and 18S V4 regions. We generate the first DNA barcode reference library for seagrass beds in Scotland to support future biomonitoring of these priority habitats.

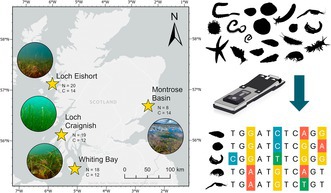

## 1 Introduction

DNA barcoding is a method for identifying species using short, diagnostic sections of their genome (Hebert et al. 2003). It was proposed as an alternative to morphological identification, with advantages including improved objective or quantitative delimitation, a reduction in processing time for large numbers of specimens, and the ability to identify organisms regardless of body size, condition, or life stage. As a result, DNA barcoding and the higher throughput (meta)barcoding have become popular and routine tools for species identification (DeSalle and Goldstein 2019; Ruppert et al. 2019). However, DNA barcodes are only effective for species identification when pre‐existing reference barcode(s) exist for the organisms in question.

Despite the major advances over the past two decades, many species still don't have reference barcodes, and, of those that do, most species have a small number collected from specimens covering only part of their geographic range (Weigand et al. 2019). Additionally, without comprehensive reference libraries, projects aiming to characterize communities will continue to underestimate diversity and produce inaccurate species inventories (Blackman et al. 2024). Therefore, there is an increasing need to populate DNA barcode reference libraries. One obstacle to reference library creation is the cost, ease, and success rate of contemporary methods such as Sanger sequencing when applied to hundreds of specimens. Additionally, DNA barcoding is prone to coamplifying nontarget DNA from contaminants, dietary remnants, or symbionts, which can result in spurious or low‐confidence basecalls, often necessitating the resequencing of numerous specimens.

Oxford Nanopore Technology (ONT) in combination with the ONTBarcoder pipeline has emerged as a promising tool for large‐scale DNA barcoding (Srivathsan et al. 2021). The sequence quality of ONT‐generated barcodes surpassed those generated by Sanger sequencing (Cuber et al. 2023; Koblmüller et al. 2024), and hundreds of specimens can be multiplexed in the same sequencing run with no need for assembly. Furthermore, ONT platforms sequence amplicons on a per‐molecule basis, which enables the separation of target and nontarget sequences that originate from contamination.

Common seagrass beds ( *Zostera marina* (Linnaeus 1753)) provide a range of important ecosystem services (Lilley and Unsworth 2014; Potouroglou et al. 2021) but have been badly degraded in recent decades (Green et al. 2021). As a result, there is an increased interest in applying routine monitoring methods to these habitats, one of which is metabarcoding‐based surveys targeting the macrobenthos (Cowart et al. 2015). The macrobenthos plays an important role in stabilizing seagrass meadow ecosystem functions (Duffy et al. 2017) and is a longstanding component of habitat quality indices (Borja et al. 2000). However, marine invertebrates are among the groups with the lowest barcode coverage (Radulovici et al. 2021; Weigand et al. 2019). With this in mind, we apply these methods to generate DNA barcodes for invertebrates inhabiting these habitats.

In this study, we use ONT R10 Flongle Flowcells in combination with ONTBarcoder2 (Srivathsan et al. 2024) to barcode 316 marine invertebrate specimens and generate a reference library for invertebrates inhabiting seagrass beds in Scotland. We target two popular metabarcoding markers, COI and 18S V4. We then assess the impact of input amplicon quantity (ng) on read recovery and investigate how this metric affects the quality of downstream consensus sequences. Finally, we examine the impact of a simple read mapping step in Geneious Prime to improve DNA barcode recovery by excluding nontarget reads prior to consensus sequence creation.

## 2 Materials and Methods

### 2.1 Sample Collection and Preliminary Identification

**FIGURE 1:**
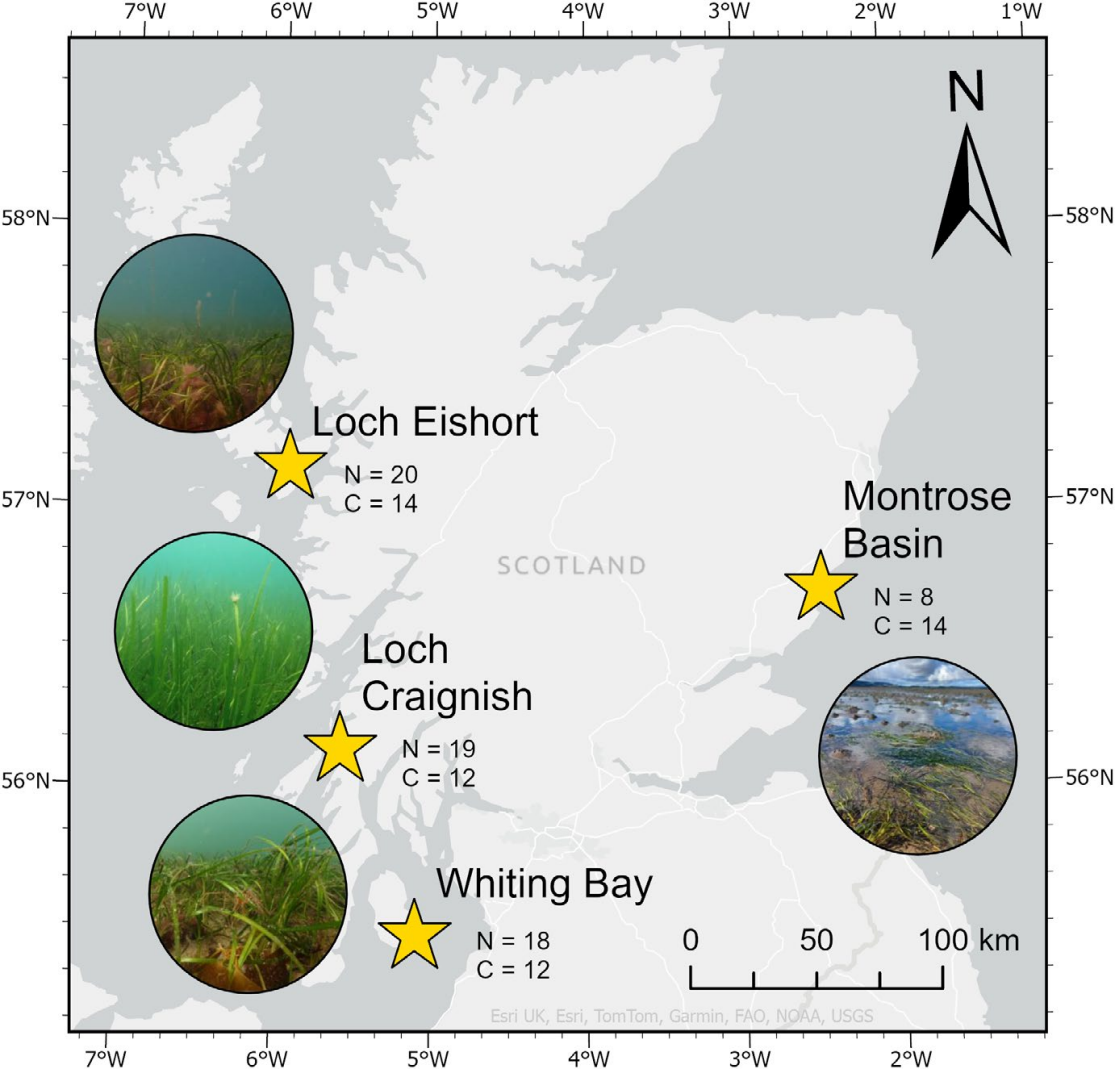
Four seagrass beds from which marine macroinvertebrates were collected Loch Craignish (56°09′17.8″ N 5°34′31.7″ W), Loch Eishort (57°08′58.3″ N 5°56′37.8″ W), Whiting Bay (55°29′22.3″ N 5°05′23.9″ W), and Montrose Basin (56°43′12.2″ N 2°28′46.2″ W) in Scotland. C = number of sediment cores collected, *N* = number of net samples collected.

Marine macroinvertebrates were collected from four sites in Scotland between December 2021 and August 2023 (Figure 1, Table S1). Epifauna were collected by passing a 500 μm mesh kick net over seagrass leaves in a sweeping motion while wading or by swimming ~3 m in a straight line over the seagrass with a 1 mm mesh, square hand‐held net. Infauna were collected in sediment cores recovered by boring 10 cm diameter, 20 cm long PVC pipes into the seabed. The sediment cores were then sieved with a 500 μm stainless steel sieve.

Next, specimens were placed in a Petri dish of seawater and photographed live using the macro settings on a Nikon COOLPIX W300 camera or a built‐in camera on a stereomicroscope (Zeiss Stemi 305). Following this, specimens were then euthanized by placing them in a Petri dish of isotonic MgCl_2_ (80 g/L) and preserved in absolute ethanol. Additional photos of ethanol‐preserved specimens were also collected using the stereomicroscope. Up to 18 specimens of each putative morphospecies per collection site were selected for processing, with a greater number of individuals selected in the presence of considerable morphological variation (e.g., rissoid gastropods). Morphological identification was then carried out by comparing each specimen to a relevant dichotomous key (Hayward and Ryland 2017). Lincoln (1979) was additionally used to identify specimens from the order Amphipoda. Identifications were made using live specimens in the field whenever possible. Otherwise, IDs were based on ethanol‐preserved specimens and their accompanying photos. Tissue voucher material was deposited at the National Museum of Scotland.

### 2.2 DNA Extraction

DNA extractions were carried out using the E.Z.N.A Tissue DNA Kit, following the manufacturer's protocol (D3396‐01 Omega Bio‐Tek). For each specimen, up to 30 mg of soft tissue was collected using sterilized forceps and a scalpel, briefly blotted with paper towel to remove excess ethanol, and placed in an Eppendorf tube with 200 μL of TL buffer (D3396‐01 Omega Bio‐Tek). Care was taken to avoid taking tissue from around the gut where dietary contaminants might be present or from tissues that could house symbionts (e.g., anemone tentacles, bivalve gills). For the dominant taxonomic groups, tissue was collected as follows: molluscs—mantle, echinoderms—tube feet, polychaetes—parapodia, and crustaceans—chela or pereopods. Between specimens, forceps and scalpels were sterilized by soaking them in 1% bleach solution, washing them in double‐distilled water. Following this, 25 μL of Proteinase K Solution (D3396‐01 Omega Bio‐Tek) was added to each tube and vortexed. All tubes were then placed in an incubator at 55°C overnight. Extractions were carried out in batches of 12–36 specimens at a time, and a negative extraction control of 25 μL of Proteinase K Solution and 200 μL of TL buffer with no added tissue was included in each batch. The remaining extraction steps followed the manufacturer's protocol. Rissoid snails extracted with the E.Z.N.A Tissue DNA Kit repeatedly failed PCR. As a result, all rissoids were instead extracted using the E.Z.N.A Mollusc DNA Kit following the manufacturer's protocol (D3373‐00S, Omega Bio‐Tek), which was effective at removing PCR‐inhibitory mucopolysaccharides. After extraction, DNA was quantified (ng/μL), and the ratio of absorbance at 260 and 280 nm was used to assess DNA quality using a nanodrop spectrophotometer.

### 2.3 PCR

A total of 316 specimens were selected for COI amplification, and 225 specimens were selected for 18S amplification. All PCRs were carried out in 25 μL reactions using the Taq PCR Kit (#E5000S; New England Biolabs) with the following composition: 10× Standard Taq Reaction Buffer 2.5 μL, 10 mM dNTPs 0.5 μL, 10 μM forward primer 0.5 μL, 10 μM reverse primer 0.5 μL, Taq DNA polymerase 0.125 μL, nuclease‐free water 18.875 μL, and undiluted template DNA 2 μL. PCR primers were tagged with index sequences (Cuber et al. 2023), with unique combinations of indices used for the amplification of each specimen.

COI PCR reactions used four tagged primer pairs LCO1490/HCO2198 (Folmer et al. 1994) (*n* = 266), jgLCO1490/jgHCO2198 (Geller et al. 2013) (*n* = 39), polyLCO/polyHCO (Carr et al. 2011) (*n* = 9) and LCOech1aF1/HCO2198 (Layton et al. 2016) (*n* = 10) (Table S2) with the following program: 120 s at 95°C, followed by 3× amplification cycles of denaturation for 40 s at 94°C, primer annealing for 40 s at 45°C and extension for 60 s at 72°C followed by 30× cycles of denaturation for 40 s at 94°C, primer annealing for 40 s at 55°C and extension for 60 s at 72°C with a final elongation step at 72°C for 5 min. All 18S PCRs were performed with Uni18S/Uni18R (Zhan et al. 2013) (Table S2) with the following PCR program: 5 min at 95°C, followed by 25× amplification cycles of denaturation for 30 s at 95°C, primer annealing for 30 s at 50°C and extension for 90 s at 72°C, followed by a final elongation step at 72°C for 10 min. All COI primer combinations shared an expected amplicon length of ~658 bp, while 18S amplicon lengths varied between 400 and 600 bp.

PCR products were then visualized on a 2% agarose gel with SYBR Safe DNA stain and a 1 kb DNA ladder (NEB Quick‐Load Purple 1 kb DNA Ladder), run for 50 min at 6 Vcm^−1^. PCR products with visible bands matching the expected amplicon length were then selected for sequencing.

### 2.4 ONT Flongle Sequencing and Consensus Sequence Generation

In total, six R10 Flongle Flowcells were used for sequencing (FLO‐FLG114; Oxford Nanopore Technologies). To create each sequencing library, amplicons were first pooled by adding 3 μL of each PCR product to a 1.5 mL Eppendorf tube. Next, the combined concentration of the pooled amplicon mixture was measured using a Qubit Fluorometer High Sensitivity Assay kit (Q32851; Thermo Fisher Scientific). 200 fmol of the pooled amplicon mixture was subsampled for library preparation. Library preparation was then carried out following the manufacturer's protocol for ligation sequencing amplicons using the V14 Ligation Sequencing Kit (SQK‐LSK114; Oxford Nanopore Technologies) and the NEBNext Companion Module (E7180S; New England Biolabs). DNA was eluted in 7 μL of elution buffer and its concentration was measured using a Qubit Fluorometer High Sensitivity Assay kit. 20 fmol DNA was then loaded into a Flongle Flow Cell (R10.4.1), and sequencing commenced over a 24‐h period.

Raw sequencing reads were basecalled using Guppy (version 23.07.15) high accuracy basecalling using the configuration file “dna_r10.4.1_e8.2_400bps_5khz_hac.cfg”, with a minimum average read Q score of 10 and adapter trimming on. The mean Q score for passed reads was then obtained using FastQC (version 0.11.9) (Andrews 2010). Passed reads were then demultiplexed and converted into consensus sequences using ONTBarcoder2 mode 1 (Srivathsan et al. 2021). The ONTBarcoder2 run was carried out with default settings for COI and modified settings for 18S (Table S3). Barcodes created by similarity were preferentially selected over those generated by length since the 90% similarity threshold was deemed useful in excluding contamination reads from the consensus sequences. Barcodes generated by length were only selected if there was no associated barcode generated by similarity. If a specimen had no consensus sequence, it was considered unrecovered. Consensus by barcode comparison barcodes were ignored in favor of a mapping process discussed below. Furthermore, ONTBarcoder2 also flagged each sequence as QC compliant if they were translatable, matched the expected amplicon length, and were free from ambiguous bases. This was recorded for COI only since 18S is not a protein coding gene. The percentage of ambiguous bases for each sequence was recorded to ensure they were in compliance with the Barcode of Life Database upload requirements, with fewer than 1% ambiguous bases across the total sequence length (Milton et al. 2013).

### 2.5 Taxonomic Assignment

**FIGURE 2:**
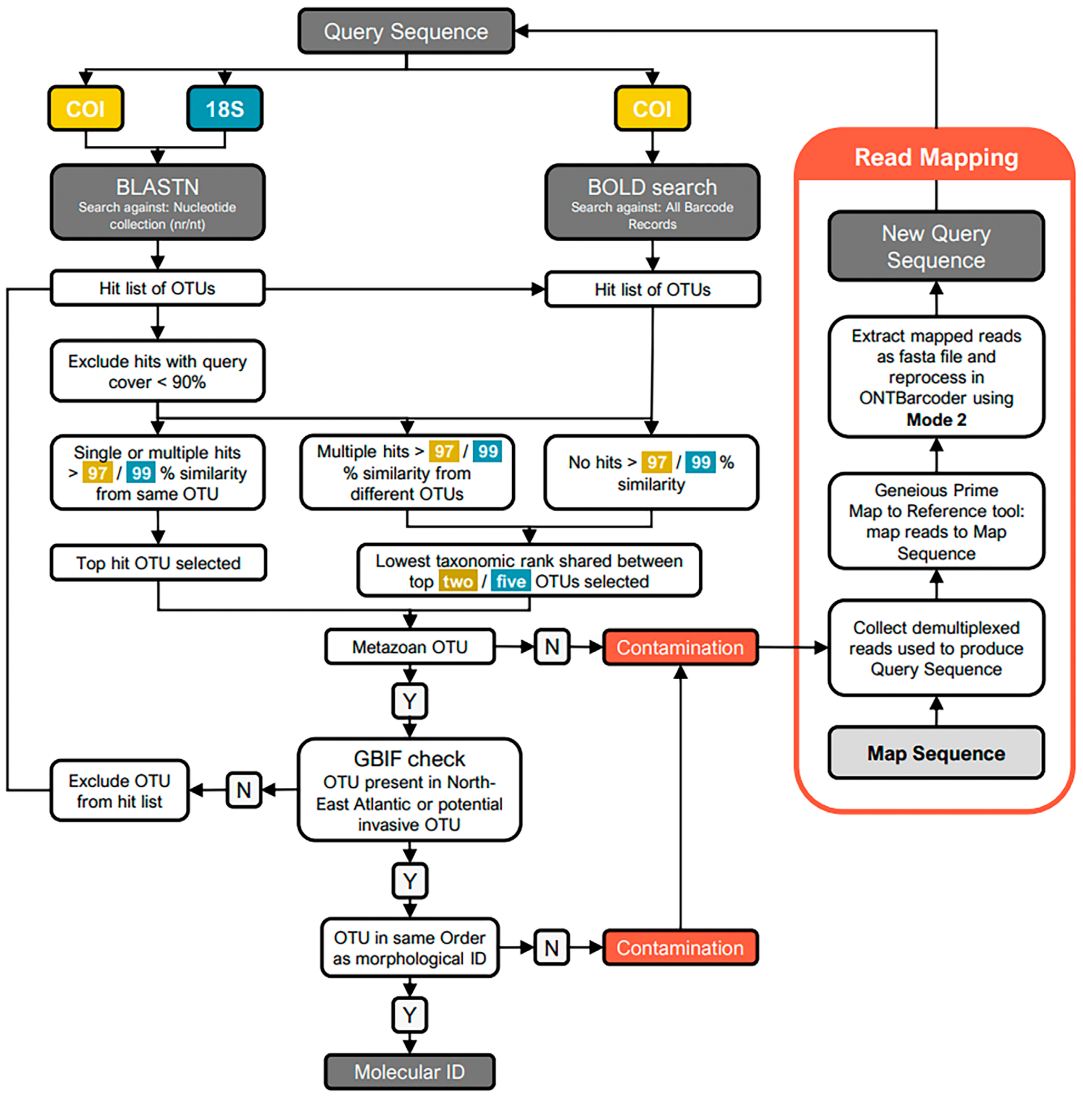
Workflow used to generate molecular IDs for marine invertebrates for two DNA barcodes: COI and 18S. N = No, Y = Yes.

Consensus sequences were then queried against the NCBI Nucleotide collection (nr/nt) (Sayers et al. 2025) using BLASTN with default settings and the Barcode of Life Database (BOLD V4) (Ratnasingham et al. 2024) using the online identification tool (Figure 2). BOLD searches were made against the full‐length record barcode database library, which included taxa identified to taxonomic levels above species. No species‐level identifications were considered unless COI hits were > 90% query cover and > 97% sequence similarity. 18S ONT sequences were queried against the NCBI Nucleotide collection (nr/nt) only (Figure 2). No species‐level identification was considered from any 18S hits, and genus‐level identifications were only considered if there were > 90% query cover and 100% sequence similarity. The identity of each hit was then checked to see if the assigned species name was valid in the World Register of Marine Species (WoRMS) (WoRMS Editorial Board 2025) and if the species was native to the North East Atlantic by manually searching the assignments on GBIF (GBIF.org 2025). We saw no evidence of invasive species. Following this, the IDs from morphology, COI, and 18S were assessed for congruence. The identification methods were considered congruent when the taxonomic rank of one of the IDs was the same or sat within the taxonomic rank of another; for example, if the 18S ID was Nereididae and the COI ID was *Platynereis dumerilii* (Audouin and Milne Edwards 1833). Final taxonomic assignments were then used to assess the number of operational taxonomic units (OTUs). Here, we consider an OTU to be a molecular and/or morphologically distinct specimen or group of similar specimens, which likely represents a single species‐level unit.

### 2.6 Mapping of Reads to Improve DNA Barcode Recovery

When ONTBarcoder2 produced barcodes with ambiguous bases and/or that belonged to a nontarget OTU, attempts were made to recover a DNA barcode with fewer ambiguities from the target OTU by mapping target reads to a preexisting DNA barcode, hereafter referred to as the map sequence (Figure 2). For each specimen, all demultiplexed reads were mapped to a map sequence using the “Map to Reference” tool in Geneious Prime, beginning with the low sensitivity/fastest setting and sequentially increasing the sensitivity by swapping between the five pre‐set mapping sensitivity options until reads were mapped. Mapping sensitivity was increased by increasing the maximum gap size, number of mismatches allowed between the reads and the map sequence, and decreasing the word lengths used in the alignment. Map sequences were selected based on the putative morphological ID of the target specimens. Map sequences were preferentially taken from noncontaminated DNA barcodes that were recovered from other specimens with the same putative morphological ID as the target from within our dataset. If there were no other specimens in our dataset that morphologically matched, a pre‐existing DNA barcode from a specimen assigned to the same rank as the putative morphological ID was collected from BOLD (COI) or GenBank (18S). If there were no preexisting entries matching the putative morphological ID, a closely related organism was selected instead (e.g., same genus or same family).

Mapped reads were then fed back into ONTBarcoder2 using mode 2 with the same consensus creation parameters as demultiplexing (Table S3). Again, barcodes created by similarity were preferentially selected over those generated by length. The new consensus sequences were then re‐queried against the public reference library as in Figure 2. The new barcode replaced the DNA barcode originally produced by ONTBarcoder2 if it belonged to the target organism and contained fewer ambiguous bases.

### 2.7 Assessing the Relationship Between Input DNA and the Number of Demultiplexed Reads

For the second Flongle run, eight specimens, each representing different species, were selected to test the impact of the amount of input amplicon DNA (ng) added into the Flongle sequencing library on the number of demultiplexed sequences recovered after sequencing by ONTBarcoder2. Barcodes for the eight species had already been sequenced in a preceding Flongle run, and specimens were selected to cover multiple phyla. PCRs for each specimen were carried out in triplicate using different combinations of tagged LCO1490/HCO2198 primers. PCRs for *Asterias rubens* (Linnaeus 1758) used primers LCOech1aF1/HCO2198 instead. The concentration of each PCR product was measured using a Qubit Fluorometer (high sensitivity). PCR products underwent a two‐step dilution process using molecular grade water to create different input concentrations from each triplicate. First, products were standardized so that those with a concentration of < 45 ng/μL were diluted to 9 ng/μL, while those ≥ 45 ng/μL were diluted to 45 ng/μL. Next, from these standardized concentrations, distinct high, medium, and low inputs were generated for each triplicate. If the starting standardized concentration was 9 ng/μL, the three inputs were 9, 2.25, and 0.5625 ng/μL. If the starting standardized concentration was 45 ng/μL, the three inputs were 45, 15, and 5 ng/μL (Table S4). This method ensured a range of input concentrations that reflected the observed variability in PCR product concentrations across the dataset. A Nanopore sequencing library was then created using the steps outlined above.

### 2.8 Plotting and Statistical Analysis

All plots were generated using the *ggplot2* R package (v3.5.1) (Wickham 2016). The seagrass map was made in ArcGIS (v3.1.2) (Esri 2025) using the light grey canvas basemap (Esri et al. 2025). A linear model was used to assess the relationship between input amplicon concentrations and the number of demultiplexed reads using the base R *lm* function from the *stats* package (v4.2.3) (R Core Team 2023). Normality of residuals and homogeneity of variance were assessed using the *plot* function.

## 3 Results

### 3.1 Generating an ONT Reference Library

Using a combination of morphology and DNA barcodes, we identified 150 OTUs, spanning 12 phyla, 24 classes, 42 orders, 92 families, and 101 genera. The four dominant metazoan groups were Polychaeta (*n* = 45), Malacostraca (*n* = 38), Gastropoda (*n* = 20), and Bivalvia (*n* = 14), collectively accounting for 78% of OTUs in the dataset. For 119 OTUs (80%), both COI and 18S DNA barcodes were recovered; for 14 OTUs (9%), only a COI barcode was recovered; for 13 OTUs (9%), only an 18S barcode was recovered; and for 4 OTUs (2%), neither barcode was recovered. Using the COI DNA barcodes and morphology on their own, the most common taxonomic rank was species, although 56% of the species‐level IDs from morphology were revoked after querying the corresponding sequences against the molecular databases (Figure S1). The most common taxonomic rank to which 18S barcodes resolved was order, closely followed by family. Excluding cases when one of the markers was contained, there was congruence between the COI ID and 18S ID for all OTUs. There was congruence between molecular IDs (COI and 18S combined) and morphological IDs for 78% of OTUs. Finally, COI barcodes were generated for six morphologically identified species that had no previous COI DNA barcodes on BOLD, including *Calliopaea bellula* d'Orbigny 1837, *Leptocheirus pectinatus* (Norman 1869), *Leucothoe spinicarpa* (Abildgaard 1789), *Moerella donacina* (Linnaeus 1758), *Praunus neglectus* (G. O. Sars 1869), and *Travisia forbesii* (Johnston 1840).

### 3.2 Tagged PCR Success and Flongle Sequencing Recovery

COI amplicons were generated for 139 OTUs (93%), and 18S amplicons were generated for 134 OTUs (90%). Without the use of the degenerate and group‐specific primers, COI barcodes for 23 OTUs (17%) would not have been recovered. There was no overlap in the OTUs that failed COI PCRs compared to those that failed 18S PCRs. In total, 362 COI and 218 18S amplicons were loaded into the MinION across six different Flongles. The number of raw reads produced by each Flongle varied between 290,701 and 426,031 and was not influenced by the number of active pores (*F*_(1,4)_ = 5.9, *p* > 0.05). The majority of reads had a mean basecall accuracy between 96.8% and 99.2% (Table 1). Approximately 50% of raw reads were successfully demultiplexed by ONTBarcoder2 in each sequencing run. COI consensus sequences were recovered for 334 amplicons (92.3%), and 18S consensus sequences were recovered for 215 amplicons (98.6%) but varied between runs. The number of COI barcodes that were QC compliant varied between 50% and 91%. Across the three runs with both 18S and COI amplicons, a higher percentage of 18S amplicons was recovered when compared to COI (Table 1).

**TABLE 1:** Flongle run summaries and sequences produced by ONTBarcoder2 prior to mapping.

Flongle run	Raw reads	Basecall Error *p*	Marker	Reads demultiplexed	Amplicons added to library	Amplicon sequences recovered	QC compliant barcodes
RUN1 (pores = 63)	290,701	3.2%	COI	142,220 (49%)	68	62 (91.1%)	51 (82%)
RUN2 (pores = 39)	321,661	3.2%	COI	170,638 (53%)	33	33 (100%)	30 (91%)
RUN3 (pores = 60)	360,194	3.2%	COI	173,142 (48%)	117	106 (90.6%)	71 (67%)
RUN4 (pores = 45)	264,578	0.8%	COI	1111 (0.4%)	13	12 (92.3%)	6 (50%)
			18S	153,528 (58%)	113	113 (100%)	—
RUN5 (pores = 50)	303,245	2.5%	COI	71,026 (23%)	79	71 (89.9%)	51 (72%)
			18S	69,020 (23%)	29	28 (96.6%)	—
RUN6 (pores = 85)	426,031	2.5%	COI	84,636 (20%)	52	50 (96.2%)	40 (80%)
			18S	128,451 (30%)	76	74 (97.4%)	—

*Note:* Pores = number of active nanopores at the beginning of the sequence run. Raw reads = the total number of reads produced by the sequencing run. Basecall Error *p* = probability of incorrect basecall based on mean read quality (Q score). Reads demultiplexed = the total number of reads successfully demultiplexed by ONTBarcoder2. QC compliant barcodes are translatable, match the target length, and are free of ambiguous bases.

### 3.3 Sequence Quality

**FIGURE 3:**
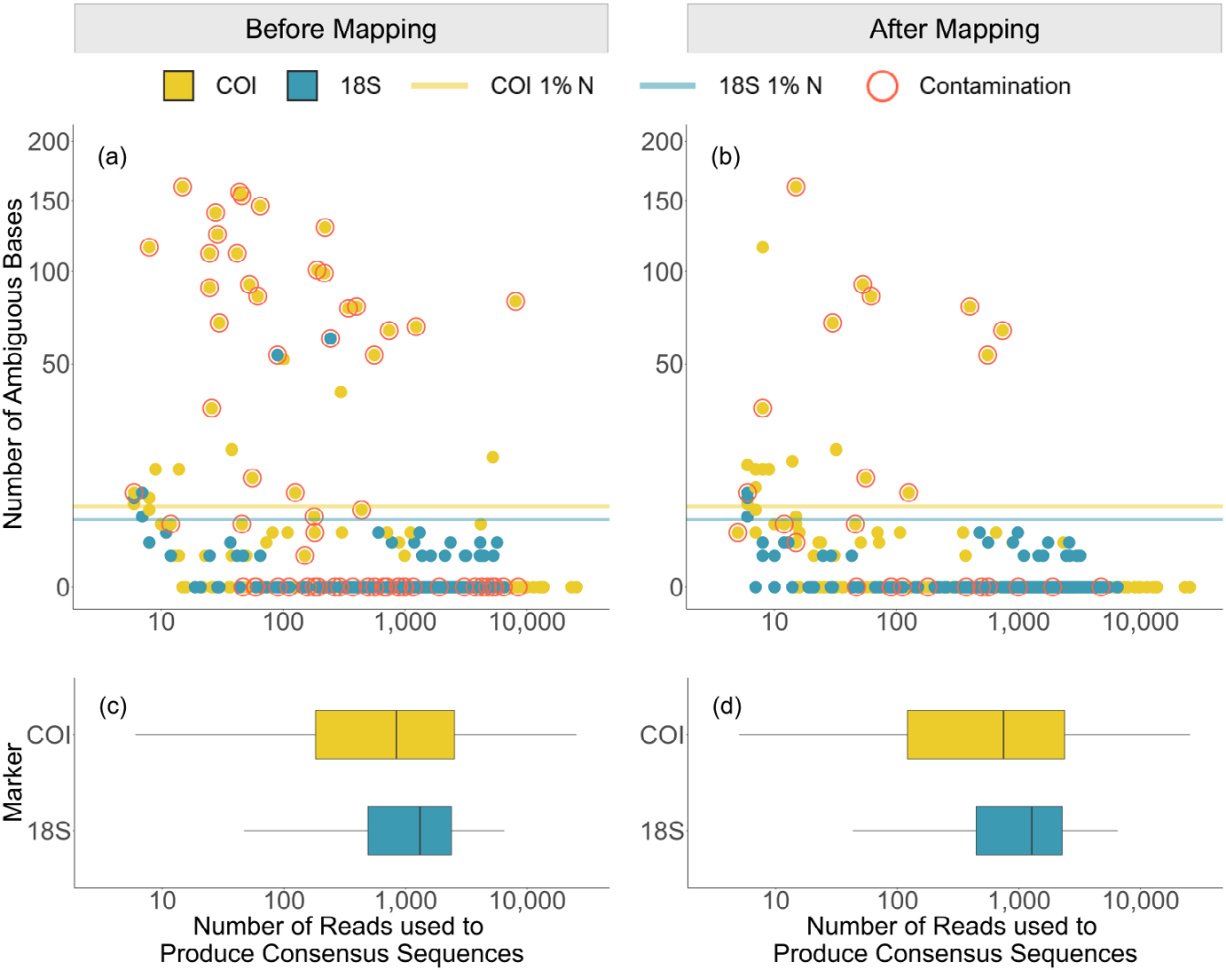
Relationship between the number of reads used to produce consensus sequences and their sequence quality before and after mapping. Scatterplots (a) and (b) show the relationship between the number of reads used by ONTBarcoder2 and the resulting number of ambiguous bases in the consensus sequences, with square root transformation applied to the *y*‐axis and log transformation applied to the *x*‐axis. COI and 18S 1% N show the threshold for greater than 1% ambiguous bases. (c) and (d) show the distribution of read counts used to produce consensus sequences, with the *x*‐axis log transformed.

After consensus sequence creation, the number of ambiguous bases in the consensus sequences decreased when higher numbers of reads were used to produce them, and the sequences with the highest number of ambiguous bases were mostly contaminated (Figure 3a). By including the mapping step, the number of ambiguous bases per sequence decreased, particularly for COI (Figure 3b). Selecting only sequences that belonged to the same OTU before and after mapping resulted in an average reduction of 2.2 ambiguous bases per sequence. The median number of reads per consensus sequence prior to mapping for COI was 861 and for 18S was 1318 (Figure 3c). After mapping, this dropped to 744 and 1274, respectively, due to nontarget reads being removed prior to creating consensus sequences (Figure 3d). Notably, some consensus sequences retained small numbers of ambiguous bases even after mapping and despite a high number of demultiplexed reads (e.g., > 1000), especially for 18S (Figure 3b).

In total, 277 COI sequences (83.2%) were all translatable and contained no ambiguous bases. 56 (16.8%) COI sequences had ambiguous bases, with 34 (10.2%) of these with ambiguous bases making up more than 1% of the total amplicon length. After including the mapping step, the number of COI sequences with ambiguous bases decreased by nine, to 47 (14.1%), and the number of these with ambiguous bases making up more than 1% of the total amplicon length dropped to 20 (6.0%). For 18S, 184 (85.6%) sequences had no ambiguous bases while 31 (14.4%) sequences had ambiguous bases, with five (2.3%) sequences with ambiguous bases greater than 1% of the total amplicon length. After the mapping step, the number of 18S sequences with ambiguous bases decreased by five to 26 (12.1%), and the number of these with ambiguous bases making up more than 1% of the total amplicon length dropped to 3 (1.4%).

**FIGURE 4:**
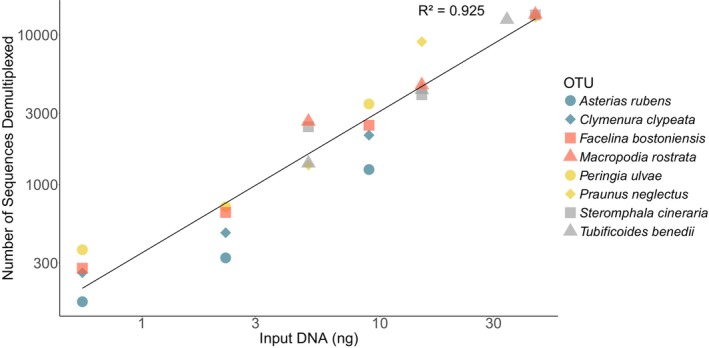
Relationship between the amount of input amplicon DNA (ng) added to an ONT R10 Flongle and the number of reads recovered. Both the *x* and *y*‐axes have been log‐transformed.

By diluting amplicons from eight specimens, a clear positive relationship emerged, showing that the amount of input amplicon DNA drives the number of demultiplexed reads recovered postsequencing (*F*_(1,22)_ = 272.6, *p* < 0.05) (Figure 4).

### 3.4 Contamination

COI consensus sequences were recovered for 286 specimens. Of these, 237 (83%) belonged to the target OTU, while 49 (17%) derived from contamination. Similarly, 18S consensus sequences were recovered for 213 specimens. Of these, 202 (95%) belonged to the target OTU, while 11 (5%) derived from contamination. By including the mapping step, it was possible to recover consensus sequences belonging to the target OTU for 31 of the 49 initially contaminated specimens (63%). This increased the recovery of consensus sequences belonging to the target OTU to 268 out of 286 specimens (94%). For 18S, it was possible to recover consensus sequences belonging to the target OTU for 7 of the 11 initially contaminated specimens (63%). This increased the recovery of noncontaminated consensus sequences to 209 out of 213 specimens (98%). The rates of contamination in Bivalvia were disproportionately higher when compared to the other dominant classes (Polychaeta, Gastropoda, and Malacostraca (*χ* ^2^_(3)_ = 10.34, *p* < 0.05)), the sources being from macroalgae or marine gammaproteobacteria. Overall, COI barcodes for 14 OTUs (10% of OTUs in the dataset) and 18S barcodes for 6 OTUs (4% of OTUs in the dataset) would not have been recovered without completing the mapping step.

## 4 Discussion

This study successfully generated a reference library of 146 marine invertebrates utilizing two DNA markers, five PCR primer pairs, and a mapping step that improved recovery for 38 OTUs that were initially contaminated.

### 4.1 PCR Success

For projects aiming to barcode species from across multiple phyla, we show that the use of group‐specific and degenerate primers can improve PCR success and can be easily incorporated into the ONTBarcoder2 pipeline. Additionally, multimarker approaches are becoming more common in DNA metabarcoding studies to account for the taxonomic PCR biases of different markers (Robinson et al. 2022; Zhang et al. 2018). In this dataset, there are several examples of OTUs amplifying with one but not both markers, and we therefore suggest that multiple marker approaches should become commonplace in reference library creation too, especially since different markers can be sequenced simultaneously using ONT. There were only four instances where neither amplicon was recovered: a demosponge and a polychaete that were contaminated, and another polychaete and an amphipod that yielded 0 ng/μL after extraction and unsurprisingly failed to amplify. Poor amplification in Porifera is well known in DNA barcoding (Vargas et al. 2012), and the small body size of the other specimens (< 3 mm) may not have yielded enough target DNA.

### 4.2 Sequence Recovery

Using R9 Flongle flowcells, Srivathsan et al. (2021) had a recovery rate between 93%–95% when sequencing between 191–257 amplicons, while Cuber et al. (2023) had a recovery rate between 51%–84% when sequencing 220 amplicons. More recently, Srivathsan et al. (2024) demonstrated a 91% and 93% recovery rate when sequencing 285 amplicons using R10 Flongle flowcells with high accuracy and superaccuracy basecalling, respectively, which is comparable to our recovery rate of 91%–100% when sequencing between 33–128 amplicons with high accuracy basecalling. The number of specimens we ran per Flongle was well below the upper limit given around 250 specimens can be sequenced per run with no decrease in recovery (Srivathsan et al. 2024). With these previous studies in mind, the change from R9 to R10 reaction chemistry seems to have increased recovery and decreased variability between individual runs.

We also saw substantial variation in demultiplexed reads by up to five orders of magnitude among amplicons. Given the strong positive relationship between input DNA and the number of recovered reads and that we did not standardize the input amplicon concentrations prior to sequencing, this variation is likely explained by unequal representation of individual amplicons in the sequencing library. To further improve recovery, standardized input concentrations could be introduced; however, in agreement with Srivathsan et al. (2024), we recognize the trade‐off in cost and time by modifying the input concentrations for hundreds of specimens, given the low cost of resequencing.

### 4.3 Mapping to Improve Barcode Recovery

Ambiguous bases were common in consensus sequences for both markers. The first likely cause of ambiguous bases is sequencing error. Although the basecalling accuracy of ONT has historically been lower than other sequencing technologies, this is not the case with current R10.4.1 reaction chemistry (Bogaerts et al. 2024). Other studies show average read accuracies of > 97%, consistent with our data (Cuber et al. 2023; Zhang et al. 2023). Using super high accuracy basecalling is also likely to improve sequence quality (Koblmüller et al. 2024; Srivathsan et al. 2024). Despite this, it is unlikely consensus sequences will be accurately resolved without an adequate number of reads. We see ambiguities are more pronounced in consensus sequences made from small numbers of reads in line with previous work (Koblmüller et al. 2024). The highest number of reads that resulted in a DNA barcode with more than 1% ambiguous bases was 32, while for an untranslatable sequence, it was 24. Therefore, we suggest a minimum threshold of 30 reads should ensure high‐quality DNA barcodes.

The second possible cause of ambiguous bases is contamination, where target and nontarget reads are erroneously merged into a single consensus. Indeed, we see that the consensus sequences with the most ambiguous bases were also frequently contaminated and mostly associated with COI versus 18S. This is not surprising given that the high rates of nonspecific amplification for “universal” COI primers are well known (Lobo et al. 2013). Poor COI amplification of target DNA may also be exacerbated by the presence of biomolecules coextracted from tissues that can inhibit PCR, such as mucopolysaccharides (Jaksch et al. 2016; Layton et al. 2014). Indeed, in this study, the Omega Bio‐Tek E.Z.N.A. Mollusc DNA Kit was necessary to remove such substances before PCR amplification was possible in rissoid snails. Additionally, poor amplification in bivalves using Folmer primers has previously been demonstrated (Barco et al. 2016; Layton et al. 2014) and likely explains the higher rate of contamination in this group. Furthermore, degenerate primers jgLCO1490/jgHCO2198 (Geller et al. 2013) were required to generate noncontaminated barcodes in half of our bivalve OTUs, further highlighting the benefits of these types of primers for problematic groups.

Finally, ambiguous bases may have been driven by legitimate intragenomic amplicon sequence variants (ASVs). In these cases, reads from different ASVs are erroneously combined into a single consensus sequence since ONTBarcoder2 expects only a single ASV per specimen. For COI, this may be due to heteroplasmy (Magnacca and Brown 2010; Rodríguez‐Pena et al. 2020), the doubly uniparental inheritance of mtDNA, like in some bivalve molluscs (Passamonti and Ghiselli 2009), or even coamplification of nuclear pseudogenes (numts) (Song et al. 2008). Notably, three of the four COI barcodes recovered from the bivalve *Fabulina fabula* (Gmelin 1791) and three of the four COI barcodes recovered from ascidian *Ascidiella aspersa* (Müller 1776) had ambiguous bases despite high numbers of demultiplexed reads, which could be evidence of these phenomena in these species. Alternatively, for 18S, nonidentical intragenomic copies of the 18S rDNA may be coamplified during PCR (Pereira et al. 2020; Wang et al. 2023). We saw some evidence in of this in 18S consensus sequences, which retained small numbers of ambiguous bases even after mapping and despite high numbers of noncontaminated reads. While users should continue to optimize PCR reactions to minimize coamplification and use stringent sampling and lab precautions, we show mapping is a worthwhile step to improve recovery and quality of DNA barcodes from contaminated specimens. However, researchers should also be aware that, in cases of coamplifying intragenomic ASVs and pseudogenes, ONTBarcoder2 will likely produce somewhat chimeric sequences, which may affect the specificity of taxonomic assignments.

Furthermore, our mapping approach depends on a priori knowledge of the taxonomy of the target specimen to identify a suitable map sequence, which may not be possible for all specimens. As an alternative, demultiplexed reads could be preclustered *de novo* before generating consensus sequences to separate target and nontarget reads using sequence similarity alone. Following this, ONTBarcoder2 could be run using reads assigned to each cluster. However, there is currently no streamlined method to do this using a GUI application, which should be a priority for future work to improve adoption.

### 4.4 Variable Length Amplicons

The length of translatable COI amplicons for many marine invertebrates deviated from the expected 658 bp, suggesting codon variation in our data, up to two codons below (e.g., *Trivia monacha* (da Costa 1778)) and eight codons above expected (e.g., *Polyophthalmus* sp.). COI amplicon length variation has been acknowledged in previous studies working on marine invertebrates (Barco et al. 2016). The 18S V4 amplicon is a noncoding region with a much higher variation in length from 421 bp ( *Cephalothrix rufifrons* ) to 741 bp (*Harpinia* sp.), notably higher than the expected upper size limit of 600 bp (Zhan et al. 2013).

Variable lengths among the COI amplicons may confound ONTBarcoder2 because the pipeline was designed for equal lengths among amplicons (default 658 bp). In the barcode fixing step, the final consensus sequence length will be modified to conform with the expected length set by the user (Srivathsan et al. 2021). Because of this caveat, we omitted the barcode fixing step of the pipeline; however, this came at the cost of legitimate errors such as small insertions or deletions not being corrected automatically. This was particularly problematic for consensus sequences produced from a small number of reads since ONT sequencing is known for elevated levels of indels (Chiou et al. 2023). Furthermore, length‐variable amplicons will not be flagged as QC compliant by ONTBarcoder2, even if they are translatable and free of ambiguities, which may lead to misleading interpretations of sequence quality. For length‐variable noncoding regions, the recommendation is to run the pipeline without the consensus by similarity and barcode fixing steps (https://github.com/asrivathsan/ONTBarcoder2). However, we found the exclusion of reads by the 90% similarity threshold helped in removing nontarget reads. Additionally, we saw no major differences in the length of the 18S barcodes produced by length and similarity.

### 4.5 Implications for the Future of DNA Barcoding

Ultimately, the intention behind using ONT for DNA barcoding is to rapidly sequence specimens at a minimal cost, with maximum ease. Indeed, ONT‐based barcoding has now surpassed the cost effectiveness of other third‐generation sequencing platforms such as the PacBio Sequel (Hebert et al. 2025). Additionally, considering the total cost of all Flongle flowcells and reagents, sequencing the same number of amplicons using bidirectional Sanger sequencing would have doubled the cost per sequence. We only used six Flongle flowcells in this study, noting that our entire dataset could have been run using only three. The ONT library preparation is intuitive, making it easy to train new users and can be carried out in a few hours. The sequencing and bioinformatics can be completed within 24 h and in real time (Srivathsan et al. 2024). Furthermore, given a powerful enough desktop computer, ONT basecalling and ONTBarcoder2 can be run locally using GUIs, without needing an HPC, which has substantial advantages for researchers without these facilities.

Irrespective of technological advancement, DNA barcodes should still be linked to correctly identified specimens before they can be used in a reference library. This issue is still pervasive in public sequence repositories (Leray et al. 2022; Weigand et al. 2019), and we argue that gathering appropriate taxonomic expertise for cross‐phyla studies is still one of the greatest challenges in this field. The majority of our identifications were done by reverse taxonomy, with over half of our morphological species IDs revoked in light of the molecular data. We were fortunate to carry out this work in a locality where invertebrate diversity is historically well known, with most of our OTUs having pre‐existing DNA barcodes from neighboring geographic regions. However, many barcoding projects will not have this luxury, and therefore a reliance on reverse taxonomy alone should not motivate reference library generation moving forward.

## 5 Conclusions

Flongle sequencing and the ONTBarcoder2 analysis pipeline represent a significant jump in the ease and scale at which in‐house DNA barcoding can be carried out, with important applications for monitoring priority habitats. This pipeline works efficiently for barcoding of marine invertebrates across 11 phyla, though users should recognize the drawbacks of using ONTBarcoder2 for length‐variable, noncoding amplicons and consider the possible influence of intragenomic sequence variants. Additionally, we show mapping reads can improve barcode quality and recovery by circumventing the impacts of some contamination. Finally, we generated COI DNA barcodes for six previously unsequenced invertebrate species inhabiting UK seagrass beds and demonstrate the ONTBarcoder2 pipeline is a useful tool in rapidly generating DNA barcode reference libraries.

## Author Contributions

**E. G. Ross:** conceptualization (lead), data curation (lead), formal analysis (lead), funding acquisition (lead), investigation (lead), methodology (lead), project administration (equal), resources (lead), software (lead), supervision (lead), validation (lead), visualization (lead), writing – original draft (lead), writing – review and editing (lead). **S. B. Piertney:** conceptualization (equal), methodology (equal), supervision (equal), writing – review and editing (equal). **J. D. Sigwart:** conceptualization (equal), investigation (equal), methodology (equal), supervision (equal), writing – review and editing (equal). **N. F. Crook:** data curation (supporting), formal analysis (supporting), investigation (supporting). **A. Moreau:** data curation (supporting), formal analysis (supporting), investigation (supporting). **K. K. S. Layton:** conceptualization (equal), funding acquisition (equal), investigation (equal), methodology (equal), supervision (equal), writing – review and editing (equal).

## Disclosure

Benefit‐Sharing: In addition to the reference library, this work has contributed to establishing a biodiversity baseline for the four seagrass beds we visited, and the results of this study have been shared with the respective NGOs. A community BioBlitz event was also carried out as part of specimen collection from the Isle of Arran with the National Museum of Scotland and Arran COAST to engage with locals about the biodiversity of their seagrass beds.

## Ethics Statement

Ethical permissions do not apply as all animals in this study are noncephalopod invertebrates as per the UK Animals (Scientific Procedures) Act 1986 Amendment Regulations.

## Conflicts of Interest

The authors declare no conflicts of interest.

## Supporting information


**Data S1:** ece372219‐sup‐0001‐supinfo.docx.

## Data Availability

All DNA barcodes are publicly available on BOLD under project code: ERSSI Seagrass‐associated invertebrates of North Eastern and Western Scotland. Tissue voucher material is deposited at the National Museum of Scotland. All data, metadata, and code can be found at https://github.com/Ethan‐G‐R/Chapter_1_seagrass_ONTB_ref_lib.

## References

[ece372219-bib-0049] Andrews, S. 2010. “FastQC: A Quality Control Tool for High Throughput Sequence Data [Online].” http://www.bioinformatics.babraham.ac.uk/projects/fastqc/.

[ece372219-bib-0001] Barco, A. , M. J. Raupach , S. Laakmann , H. Neumann , and T. Knebelsberger . 2016. “Identification of North Sea Molluscs With DNA Barcoding.” Molecular Ecology Resources 16: 288–297. 10.1111/1755-0998.12440. 26095230

[ece372219-bib-0002] Blackman, R. , M. Couton , F. Keck , et al. 2024. “Environmental DNA: The Next Chapter.” Molecular Ecology 33: e17355. 10.1111/mec.17355. 38624076

[ece372219-bib-0003] Bogaerts, B. , A. Van den Bossche , B. Verhaegen , et al. 2024. “Closing the Gap: Oxford Nanopore Technologies R10 Sequencing Allows Comparable Results to Illumina Sequencing for SNP‐Based Outbreak Investigation of Bacterial Pathogens.” Journal of Clinical Microbiology 62: e0157623. 10.1128/jcm.01576-23. 38441926 PMC11077942

[ece372219-bib-0004] Borja, A. , J. Franco , and V. Pérez . 2000. “A Marine Biotic Index to Establish the Ecological Quality of Soft‐Bottom Benthos Within European Estuarine and Coastal Environments.” Marine Pollution Bulletin 40: 1100–1114. 10.1016/S0025-326X(00)00061-8.

[ece372219-bib-0047] Carr, C. M. , S. M. Hardy , T. M. Brown , T. A. Macdonald , and P. D. N. Hebert . 2011. “A Tri‐Oceanic Perspective: DNA Barcoding Reveals Geographic Structure and Cryptic Diversity in Canadian Polychaetes.” PLoS One 6: e22232. 10.1371/journal.pone.0022232. 21829451 PMC3136506

[ece372219-bib-0005] Chiou, C.‐S. , B.‐H. Chen , Y.‐W. Wang , N.‐T. Kuo , C.‐H. Chang , and Y.‐T. Huang . 2023. “Correcting Modification‐Mediated Errors in Nanopore Sequencing by Nucleotide Demodification and Reference‐Based Correction.” Communications Biology 6: 1–9. 10.1038/s42003-023-05605-4. 38030695 PMC10687267

[ece372219-bib-0006] Cowart, D. A. , M. Pinheiro , O. Mouchel , et al. 2015. “Metabarcoding Is Powerful Yet Still Blind: A Comparative Analysis of Morphological and Molecular Surveys of Seagrass Communities.” PLoS One 10: e0117562. 10.1371/journal.pone.0117562. 25668035 PMC4323199

[ece372219-bib-0007] Cuber, P. , D. Chooneea , C. Geeves , et al. 2023. “Comparing the Accuracy and Efficiency of Third Generation Sequencing Technologies, Oxford Nanopore Technologies, and Pacific Biosciences, for DNA Barcode Sequencing Applications.” Ecological Genetics and Genomics 28: 100181. 10.1016/j.egg.2023.100181.

[ece372219-bib-0053] DeSalle, R. , and P. Goldstein . 2019. “Review and Interpretation of Trends in DNA Barcoding.” Frontiers in Ecology and Evolution 7: 302. 10.3389/fevo.2019.00302.

[ece372219-bib-0045] Duffy, J. E. , C. M. Godwin , and B. J. Cardinale . 2017. “Biodiversity Effects in the Wild are Common and as Strong as Key Drivers of Productivity.” Nature 549: 261–264. 10.1038/nature23886. 28869964

[ece372219-bib-0009] Esri . 2025. “ArcGIS Pro.”

[ece372219-bib-0010] Esri , TomTom , Garmin , et al. 2025. “Light Gray Canvas.”

[ece372219-bib-0011] Folmer, O. , M. Black , W. Hoeh , R. Lutz , and R. Vrijenhoek . 1994. “DNA Primers for Amplification of Mitochondrial Cytochrome c Oxidase Subunit I From Diverse Metazoan Invertebrates.” Molecular Marine Biology and Biotechnology 3: 294–299. 7881515

[ece372219-bib-0012] GBIF.org . 2025. “GBIF Home Page.” https://www.gbif.org.

[ece372219-bib-0013] Geller, J. , C. Meyer , M. Parker , and H. Hawk . 2013. “Redesign of PCR Primers for Mitochondrial Cytochrome c Oxidase Subunit I for Marine Invertebrates and Application in All‐Taxa Biotic Surveys.” Molecular Ecology Resources 13: 851–861. 10.1111/1755-0998.12138. 23848937

[ece372219-bib-0014] Green, A. E. , R. K. F. Unsworth , M. A. Chadwick , and P. J. S. Jones . 2021. “Historical Analysis Exposes Catastrophic Seagrass Loss for the United Kingdom.” Frontiers in Plant Science 12: 629962. 33747011 10.3389/fpls.2021.629962 PMC7970192

[ece372219-bib-0015] Hayward, P. J. , and J. S. Ryland . 2017. “The Marine Environment of North‐West Europe.” In Handbook of the Marine Fauna of North‐West Europe. Oxford University Press. 10.1093/acprof:oso/9780199549443.003.0001.

[ece372219-bib-0016] Hebert, P. D. N. , A. Cywinska , S. L. Ball , and J. R. deWaard . 2003. “Biological Identifications Through DNA Barcodes.” Proceedings of the Royal Society of London, Series B: Biological Sciences 270: 313–321. 10.1098/rspb.2002.2218. PMC1691236 12614582

[ece372219-bib-0017] Hebert, P. D. N. , R. Floyd , S. Jafarpour , and S. W. J. Prosser . 2025. “Barcode 100K Specimens: In a Single Nanopore Run.” Molecular Ecology Resources 25: e14028. 10.1111/1755-0998.14028. 39387679 PMC11646297

[ece372219-bib-0019] Jaksch, K. , A. Eschner , T. V. Rintelen , and E. Haring . 2016. “DNA Analysis of Molluscs From a Museum Wet Collection: A Comparison of Different Extraction Methods.” BMC Research Notes 9: 348. 10.1186/s13104-016-2147-7. 27430899 PMC4950716

[ece372219-bib-0020] Koblmüller, S. , P. Resl , N. Klar , H. Bauer , L. Zangl , and C. Hahn . 2024. “DNA Barcoding for Species Identification of Moss‐Dwelling Invertebrates: Performance of Nanopore Sequencing and Coverage in Reference Database.” Diversity 16: 196. 10.3390/d16040196.

[ece372219-bib-0048] Layton, K. , E. Corstorphine , and P. Hebert . 2016. “Exploring Canadian Echinoderm Diversity through DNA Barcodes.” PLoS One 11. 10.1371/journal.pone.0166118. PMC5117606 27870868

[ece372219-bib-0021] Layton, K. K. S. , A. L. Martel , and P. D. Hebert . 2014. “Patterns of DNA Barcode Variation in Canadian Marine Molluscs.” PLoS One 9: e95003. 10.1371/journal.pone.0095003. 24743320 PMC3990619

[ece372219-bib-0022] Leray, M. , N. Knowlton , and R. J. Machida . 2022. “MIDORI2: A Collection of Quality Controlled, Preformatted, and Regularly Updated Reference Databases for Taxonomic Assignment of Eukaryotic Mitochondrial Sequences.” Environmental DNA 4: 894–907. 10.1002/edn3.303.

[ece372219-bib-0023] Lilley, R. J. , and R. K. F. Unsworth . 2014. “Atlantic Cod (*Gadus morhua*) Benefits From the Availability of Seagrass (*Zostera marina*) Nursery Habitat.” Global Ecology and Conservation 2: 367–377. 10.1016/j.gecco.2014.10.002.

[ece372219-bib-0046] Lincoln, R. J. 1979. British marine amphipoda, Gammaridea. British Museum (Natural History).

[ece372219-bib-0024] Lobo, J. , P. M. Costa , M. A. Teixeira , M. S. Ferreira , M. H. Costa , and F. O. Costa . 2013. “Enhanced Primers for Amplification of DNA Barcodes From a Broad Range of Marine Metazoans.” BMC Ecology 13: 34. 10.1186/1472-6785-13-34. 24020880 PMC3846737

[ece372219-bib-0025] Magnacca, K. N. , and M. J. Brown . 2010. “Mitochondrial Heteroplasmy and DNA Barcoding in Hawaiian Hylaeus (Nesoprosopis) Bees (Hymenoptera: Colletidae).” BMC Evolutionary Biology 10: 174. 10.1186/1471-2148-10-174. 20540728 PMC2891727

[ece372219-bib-0026] Milton, M. , P. Pierossi , and S. Ratnasingham . 2013. Barcode of Life Data Systems Handbook. Biodiversity Institute of Ontario.

[ece372219-bib-0027] Passamonti, M. , and F. Ghiselli . 2009. “Doubly Uniparental Inheritance: Two Mitochondrial Genomes, One Precious Model for Organelle DNA Inheritance and Evolution.” DNA and Cell Biology 28: 79–89. 10.1089/dna.2008.0807. 19196051

[ece372219-bib-0051] Pereira, T. J. , A. De Santiago , T. Schuelke , S. M. Hardy , and H. M. Bik . 2020. “The Impact of Intragenomic rRNA Variation on Metabarcoding‐Derived Diversity Estimates: A Case Study From Marine Nematodes.” Environmental DNA 2: e77. 10.1002/edn3.77.

[ece372219-bib-0028] Potouroglou, M. , D. Whitlock , L. Milatovic , et al. 2021. “The Sediment Carbon Stocks of Intertidal Seagrass Meadows in Scotland.” Estuarine, Coastal and Shelf Science 258: 107442. 10.1016/j.ecss.2021.107442.

[ece372219-bib-0029] R Core Team . 2023. “R: A Language and Environment for Statistical Computing.” R Foundation for Statistical Computing: Vienna, Austria.

[ece372219-bib-0030] Radulovici, A. E. , P. E. Vieira , S. Duarte , et al. 2021. “Revision and Annotation of DNA Barcode Records for Marine Invertebrates: Report of the 8th iBOL Conference Hackathon.” Metabarcoding and Metagenomics 5: e67862. 10.3897/mbmg.5.67862.

[ece372219-bib-0031] Ratnasingham, S. , C. Wei , D. Chan , et al. 2024. “BOLD v4: A Centralized Bioinformatics Platform for DNA‐Based Biodiversity Data.” Methods in Molecular Biology 2744: 403–441. 10.1007/978-1-0716-3581-0_26. 38683334

[ece372219-bib-0032] Robinson, C. V. , T. M. Porter , K. M. McGee , M. McCusker , M. T. G. Wright , and M. Hajibabaei . 2022. “Multi‐Marker DNA Metabarcoding Detects Suites of Environmental Gradients From an Urban Harbour.” Scientific Reports 12: 10556. 10.1038/s41598-022-13262-6. 35732669 PMC9217803

[ece372219-bib-0033] Rodríguez‐Pena, E. , P. Verísimo , L. Fernández , A. González‐Tizón , C. Bárcena , and A. Martínez‐Lage . 2020. “High Incidence of Heteroplasmy in the mtDNA of a Natural Population of the Spider Crab Maja Brachydactyla.” PLoS One 15: e0230243. 10.1371/journal.pone.0230243. 32191743 PMC7082002

[ece372219-bib-0034] Ruppert, K. M. , R. J. Kline , and M. S. Rahman . 2019. “Past, Present, and Future Perspectives of Environmental DNA (eDNA) Metabarcoding: A Systematic Review in Methods, Monitoring, and Applications of Global eDNA.” Global Ecology and Conservation 17: e00547. 10.1016/j.gecco.2019.e00547.

[ece372219-bib-0035] Sayers, E. W. , J. Beck , E. E. Bolton , et al. 2025. “Database Resources of the National Center for Biotechnology Information in 2025.” Nucleic Acids Research 53: D20–D29. 10.1093/nar/gkae979. 39526373 PMC11701734

[ece372219-bib-0036] Song, H. , J. E. Buhay , M. F. Whiting , and K. A. Crandall . 2008. “Many Species in One: DNA Barcoding Overestimates the Number of Species When Nuclear Mitochondrial Pseudogenes Are Coamplified.” Proceedings of the National Academy of Sciences of the United States of America 105: 13486–13491. 10.1073/pnas.0803076105. 18757756 PMC2527351

[ece372219-bib-0037] Srivathsan, A. , V. Feng , D. Suárez , B. Emerson , and R. Meier . 2024. “ONTbarcoder 2.0: Rapid Species Discovery and Identification With Real‐Time Barcoding Facilitated by Oxford Nanopore R10.4.” Cladistics 40: 192–203. 10.1111/cla.12566. 38041646

[ece372219-bib-0038] Srivathsan, A. , L. Lee , K. Katoh , et al. 2021. “ONTbarcoder and MinION Barcodes Aid Biodiversity Discovery and Identification by Everyone, for Everyone.” BMC Biology 19: 217. 10.1186/s12915-021-01141-x. 34587965 PMC8479912

[ece372219-bib-0050] Vargas, S. , A. Schuster , K. Sacher , et al. 2012. “Barcoding Sponges: An Overview Based on Comprehensive Sampling.” PLoS One 7: e39345. 10.1371/journal.pone.0039345. 22802937 PMC3389008

[ece372219-bib-0052] Wang, W. , X. Zhang , S. Garcia , A. R. Leitch , and A. Kovařík . 2023. “Intragenomic rDNA Variation – the Product of Concerted Evolution, Mutation, or Something in Between?” Heredity 131: 179–188. 10.1038/s41437-023-00634-5. 37402824 PMC10462631

[ece372219-bib-0039] Weigand, H. , A. J. Beermann , F. Čiampor , et al. 2019. “DNA Barcode Reference Libraries for the Monitoring of Aquatic Biota in Europe: Gap‐Analysis and Recommendations for Future Work.” Science of the Total Environment 678: 499–524. 10.1016/j.scitotenv.2019.04.247. 31077928

[ece372219-bib-0040] Wickham, H. 2016. ggplot2: Elegant Graphics for Data Analysis. Springer‐Verlag New York.

[ece372219-bib-0041] WoRMS Editorial Board . 2025. “World Register of Marine Species.” https://www.marinespecies.org.

[ece372219-bib-0042] Zhan, A. , M. Hulák , F. Sylvester , et al. 2013. “High Sensitivity of 454 Pyrosequencing for Detection of Rare Species in Aquatic Communities.” Methods in Ecology and Evolution 4: 558–565. 10.1111/2041-210X.12037.

[ece372219-bib-0043] Zhang, G. K. , F. J. J. Chain , C. L. Abbott , and M. E. Cristescu . 2018. “Metabarcoding Using Multiplexed Markers Increases Species Detection in Complex Zooplankton Communities.” Evolutionary Applications 11: 1901–1914. 10.1111/eva.12694. 30459837 PMC6231476

[ece372219-bib-0044] Zhang, T. , H. Li , S. Ma , et al. 2023. “The Newest Oxford Nanopore R10.4.1 Full‐Length 16S rRNA Sequencing Enables the Accurate Resolution of Species‐Level Microbial Community Profiling.” Applied and Environmental Microbiology 89: e00605‐23. 10.1128/aem.00605-23. 37800969 PMC10617388

