# Supplementary material for: A Comprehensive DNA Barcode Reference Library for the Macroinvertebrates of Scottish Seagrass Beds Using Oxford Nanopore Flongle Flowcells
Source: Ecol Evol. 2025 Sep 28;15(10):e72219. doi: 10.1002/ece3.72219 (PMC12476923; doi:10.1002/ece3.72219)
Supplement: Supplementary file 1 — Data S1: ece372219‐sup‐0001‐supinfo.docx. [file ECE3-15-e72219-s001.docx]

A comprehensive DNA barcode reference library for the macroinvertebrates of Scottish seagrass beds using Oxford Nanopore Flongle Flowcells supplementary

**Supplementary Table 1** – Collection information for seagrass invertebrates

| Site | Collection Dates | Depth | Method of access |
| --- | --- | --- | --- |
| Loch Craignish (LC) | 04.12.21 | 1 – 2 m (Subtidal) | Wading |
| Loch Craignish (LC2) | 03.07.23 – 07.07.23 | 1 – 5 m (Subtidal) | Snorkelling |
| Loch Eishort (LE) | 15.07.22 – 16.07.23 | 1 – 2 m (Subtidal) | Wading |
| Loch Eishort (LE2) | 28.08.23 – 30.08.23 | 1 – 5 m (Subtidal) | Snorkelling |
| Whiting Bay (WB) | 19.06.23 – 21.06.23 | 4 – 8 m (Subtidal) | SCUBA |
| Montrose Basin (MB) | 09.09.22 – 12.09.22 | 0 m (Intertidal) | Wading |

**Supplementary Table 2** – Tagged PCR primers used to generate ONT DNA barcodes. XXXXXXXXXXXXX represents the unique index sequences at the 5’ end of each primer

| Target | Forward Primer | Sequence 5’ – 3’ | | Reverse Primer | | Sequence 5’ – 3’ | |  |
| --- | --- | --- | --- | --- | --- | --- | --- | --- |
| COI | LCO1490 (Folmer 1994) | | XXXXXXXXXXXXX-GGTCAACAAATCATAAAGATATTGG | | HCO2198  (Folmer 1994) | | XXXXXXXXXXXXX-TAAACTTCAGGGTGACCAAAAAATCA | |
| COI | LCOech1aF1 (Layton et al. 2016) | | XXXXXXXXXXXXX-  TTTTTTCTACTAAACACAAGGATATTGG | | HCO2198  (Folmer 1994) | | XXXXXXXXXXXXX-TAAACTTCAGGGTGACCAAAAAATCA | |
| COI | jgLCO1490 (Geller et al. 2013) | | XXXXXXXXXXXXX-TITCIACIAAYCAYAARGAYATTGG | | jgHCO2198  (Geller et al. 2013) | | XXXXXXXXXXXXX-TAIACYTCIGGRTGICCRAARAAYCA | |
| COI | polyLCO (Carr et al., 2011) | | XXXXXXXXXXXXX-GAYTATWTTCAACAAATCATAAAGATATTGG | | polyHCO (Carr et al., 2011) | | XXXXXXXXXXXXX-TAMACTTCWGGGTGACCAAARAATCA | |
| 18S (V4) | Uni18S  (Zhan et al. 2013) | | XXXXXXXXXXXXX-  AGGGCAAKYCTGGTGCCAGC | | Uni18S  (Zhan et al. 2013) | | XXXXXXXXXXXXX-GRCGGTATCTRATCGYCTT | |

**Supplementary Table 3** – ONTBarcoder parameters for generating COI and 18S consensus sequences using ONTBarcoder

| ONTBarcoder settings | COI | 18S |
| --- | --- | --- |
| Minimum Length | 658 | 350 |
| Length of barcode | 658 | 550 |
| Window to define product length (+/-) | 100 | 200 |
| Window for primer and tag search | 100 | 100 |
| Consensus by length coverage used | 25, 50, 100 ,200, 500 | 25, 50, 100 ,200, 500 |
| Maximum deviation of read length from barcode length | 50 | 200 |
| Consensus by similarity coverage used | 100 | 100 |
| Main consensus calling frequency | 0.3 | 0.3 |
| Range of frequencies to assess | 0.2, 0.5 | 0.2, 0.5 |
| Examined at step size of | 0.05 | 0.05 |
| Genetic code | Invertebrate Mitochondrial | Invertebrate Mitochondrial |
| Steps to run | Consensus by length  Consensus by Similarity | Consensus by length  Consensus by Similarity |

**Supplementary Table 4** – Input DNA quantity to Flongle run to assess impact of DNA quantity on DNA barcode quality

| SpecimenID | OTU | DNA input to library (ng) |
| --- | --- | --- |
| 034_LE_ech9_1 | Asterias rubens | 2.25 |
| 034_LE_ech9_2 | Asterias rubens | 9 |
| 034_LE_ech9_3 | Asterias rubens | 0.5625 |
| 042_LE_2_1 | Clymenura clypeata | 2.25 |
| 042_LE_2_2 | Clymenura clypeata | 9 |
| 042_LE_2_3 | Clymenura clypeata | 0.5625 |
| 027_LC_3_1 | Praunus neglectus | 45 |
| 027_LC_3_2 | Praunus neglectus | 5.01 |
| 027_LC_3_3 | Praunus neglectus | 15 |
| B013_12_MB_4_1 | Peringia ulvae | 2.25 |
| B013_12_MB_4_2 | Peringia ulvae | 9 |
| B013_12_MB_4_3 | Peringia ulvae | 0.5625 |
| 023_LC_5_1 | Facelina bostoniensis | 9 |
| 023_LC_5_2 | Facelina bostoniensis | 2.25 |
| 023_LC_5_3 | Facelina bostoniensis | 0.5625 |
| 012_LC_6_1 | Steromphala cineraria | 15 |
| 012_LC_6_2 | Steromphala cineraria | 45 |
| 012_LC_6_3 | Steromphala cineraria | 5.01 |
| B019_10_MB_7_1 | Tubificoides benedii | 5.01 |
| B019_10_MB_7_2 | Tubificoides benedii | 15 |
| B019_10_MB | Tubificoides benedii | 34.2 |
| 035_LC_8_1 | Macropodia rostrata | 45 |
| 035_LC_8_2 | Macropodia rostrata | 15 |
| 035_LC_8_3 | Macropodia rostrata | 5.01 |
| A013_10_MB | Crangon crangon | 1.305 |
| 008_LE_15_7 | Steromphala cineraria | 58.8 |
| B032_9_MB | Scoloplos armiger | 0 |
| B012_11_MB | Pygospio elegans | 0 |
| 026_LC_4_12 | Idotea baltica | 15 |
| 022_LC_4_12 | Platyhelminthes | 9.57 |
| A004_9_MB | Littorina saxatilis | 2.94 |
| 005_LE_15_7 | Bittium reticulatum | 29.64 |
| 023_LE_15_7 | Lysianassidae | 18.96 |


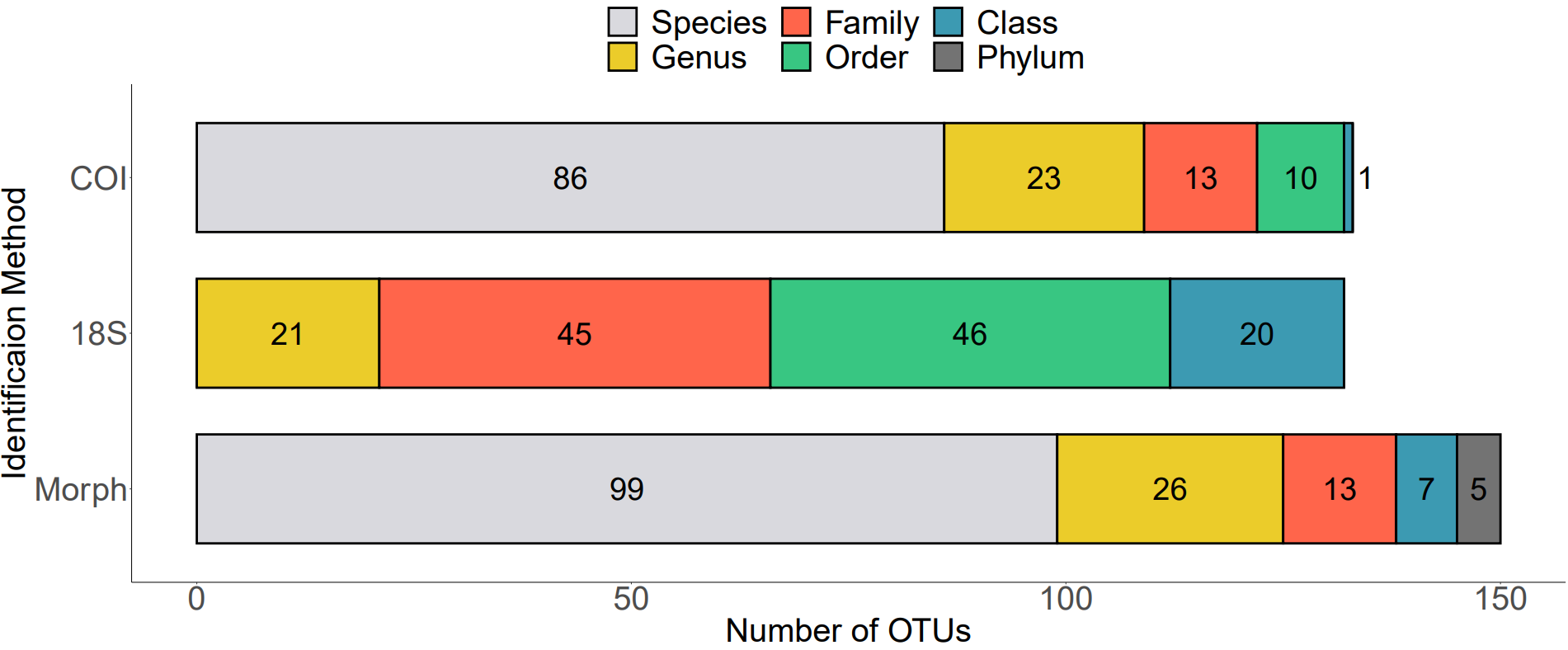


**Supplementary Figure 1** – Number of OTUs identified to different taxonomic ranks using three different methods, two DNA barcodes: COI and 18S and Morphology (Morph).
